# Ganhai Weikang Capsules ameliorate functional dyspepsia in rats by modulating gut microbiota and host metabolic homeostasis

**DOI:** 10.3389/fphar.2026.1872809

**Published:** 2026-07-30

**Authors:** Yan Rong, Liang Li, Hai-Yu Zhao, Hong-Ping Hou, Lu-Lu Jing, Yan Zhang, Li-Hua Chen, Hui-Jun Wang, Lian-Mei Wang, Ge-Jing De, Yun-Xin Chen, Li Lin, Yang Pei, Xiao-Lu Wei

**Affiliations:** 1 State Key Laboratory for Quality Assurance and Sustainable Use of Dao-di Herbs, Institute of Chinese Materia Medica, China Academy of Chinese Medical Sciences, Beijing, China; 2 School of Light Industry Science and Engineering, Beijing Technology and Business University, Beijing, China

**Keywords:** functional dyspepsia, ganhai weikang capsules (GHWKCs), gastrointestinal motility, gut microbiota, inflammation, metabolomics

## Abstract

**Background:**

Functional dyspepsia (FD) is a prevalent gastrointestinal disorder with limited long-term efficacy of conventional treatments. Ganhai Weikang Capsules (GHWKCs), a classic traditional Chinese medicine formula, has shown remarkable clinical efficacy in alleviating FD symptoms, but its underlying mechanisms, particularly on the gut microbiota-host metabolism axis, remain unclear.

**Methods:**

We performed chemical profiling of GHWKCs using UPLC-LTQ-Orbitrap-MS and established an FD rat model mimicking the TCM pathogenesis of “spleen deficiency and qi stagnation”. We evaluated gastrointestinal motility, histopathology, serum gastrointestinal hormones, and tissue inflammatory factors. Integrated duodenal metabolomics, cecal 16S rRNA sequencing, and network pharmacology were applied to explore the regulatory mechanisms, with Spearman correlation analysis conducted between differential metabolites and gut microbiota.

**Results:**

A total of 63 chemical metabolites were identified in GHWKCs. GHWKCs significantly improved gastric emptying and intestinal propulsion, normalized gastrointestinal hormone levels, and inhibited pro-inflammatory cytokine secretion in FD rats. Metabolomics revealed 18 differential metabolites mainly involved in fatty acid β-oxidation and primary bile acid biosynthesis. Gut microbiota analysis showed GHWKCs counteracted dysbiosis, particularly increasing *Coriobacteriales* and *Actinobacteriota* abundance. Correlation analysis confirmed a positive correlation between *Coriobacteriales* and glycocholic acid. Network pharmacology identified 31 active metabolites, 213 intersection targets, and key pathways including PI3K-Akt, MAPK, and Toll-like receptor signaling.

**Conclusion:**

GHWKCs ameliorate FD through multi-target mechanisms involving improved motility, reduced inflammation, and restored microbiota-metabolic homeostasis. The Coriobacteriales-glycocholic acid axis represents a putative core regulatory pathway bridging TCM theory and modern molecular mechanisms, providing a preclinical mechanistic framework for TCM-based therapy of FD.

## 1 Introduction

Functional dyspepsia (FD) is a clinically common functional gastrointestinal disorder, primarily involving the stomach and duodenum. Its typical symptoms include epigastric pain, epigastric burning, postprandial fullness, or early satiety, which severely impair patients’ quality of life and impose a substantial socioeconomic burden globally (Ford et al., 2020; Sayuk and Gyawali, 2020). The pathogenesis of FD is complex, with current evidence suggesting associations with gastrointestinal motility disturbances, visceral hypersensitivity, *helicobacter pylori* infection, and psychological factors including anxiety and depression (Xiao et al., 2021). Notably, impaired gastrointestinal motility represents a key pathophysiological mechanism observed in a significant proportion of FD patients (Koh et al., 2025).

In clinical practice, western medicine primarily focuses on relieving symptoms. Therapeutic agents include proton pump inhibitors (Moayyedi et al., 2017), prokinetic agents (Tack and Camilleri, 2018), and neuromodulators such as 5-HT receptor agonists/antagonists (Törnblom and Drossman, 2018). While these medications can alleviate symptoms in the short term, they are associated with high recurrence rates and potential side effects, creating an urgent need for novel therapeutic approaches. Traditional Chinese medicine (TCM) has a 2,000-year history of treating gastrointestinal disorders. FD is classified into TCM categories such as “epigastric pain” and “abdominal distension”, with “spleen deficiency and *qi* stagnation” and “dysfunction of stomach qi descending” recognized as the core pathogenesis (Zhang et al., 2025). Ganhai Weikang Capsules (GHWKCs) is a modern TCM formula derived from classical prescriptions, composed of eight medicinal herbs: Atractylodes macrocephala (Baizhu) for invigorating the spleen and replenishing qi, Glycyrrhiza uralensis (Gancao) for harmonizing the middle energizer and relieving pain, Sepiella maindroni (Haipiaoxiao) for astringing to stop bleeding and relieving hyperacidity, Gynostemma pentaphyllum (Jiaogulan) for tonifying *qi* and invigorating the spleen, Corydalis yanhusuo (Yanhusuo) for promoting blood circulation and relieving pain, Aurantii Fructus Immaturus (Zhishi) for breaking *qi* and eliminating distension, Phellodendron chinense (Huanbo) for clearing heat and drying dampness, and Hippophae rhamnoides (Shaji) for invigorating the spleen and promoting digestion. This formula has been widely used in clinical practice for decades and has shown significant efficacy in treating epigastric pain, protecting gastric mucosa, improving *Helicobacter pylori* eradication rates, and reducing inflammatory responses (Yuan et al., 2024). However, most previous studies have focused on clinical observations, and the underlying molecular mechanisms, particularly the regulatory effects on the gut microbiota-host metabolism axis, remain unclear.

Multiple studies have confirmed that intestinal flora dysbiosis is closely associated with the occurrence and progression of FD. Intestinal flora analysis through deep sequencing technology can reveal the connection between flora structural disorders and diseases (Tziatzios et al., 2020), while metabolomics technology can comprehensively capture dynamic changes of metabolites in organisms (Gao et al., 2024). Therefore, this study systematically identified the chemical constituents of GHWKCs and evaluated its therapeutic efficacy in an FD rat model that mimics the TCM pathogenesis of spleen deficiency and qi stagnation. By integrating metabolomics, gut microbiota analysis, and network pharmacology, we aimed to elucidate the multi-target regulatory mechanisms of GHWKCs against FD, providing a preclinical scientific basis for its clinical application and promoting the modernization of TCM.

## 2 Materials and methods

### 2.1 Experimental animals, chemicals and reagent

Forty male SPF-grade Sprague Dawley rats, aged 8–10 weeks and weighing 220–250 g, were purchased from Beijing Vital River Laboratory Animal Technology Co., Ltd (License No. SCXK (Jing)2021–0006). The rats were maintained at a temperature range of 22∼25 °C, relative humidity of 45–60%, and a 12 h light/12 h dark cycle. This study was approved by the Animal Ethics Committee of the Institute of Chinese Materia Medica, China Academy of Chinese Medical Sciences (Approval No. 2024B286) and performed in strict accordance with the 3R principles.

GHWKCs dried extract (Batch No. 2407071) was provided by Shaanxi Dongke Pharmaceutical Co., Ltd (Shaanxi, China); Mosapride citrate tablets (positive control, YaBao Pharmaceutical Group Co., Ltd., Batch No. 240312); Acetonitrile (MS-grade, Thermo Fisher Scientific, Waltham, MA, USA); Methanol (MS-grade, Shanghai Xingke High Purity Solvent Co., Ltd.); ACQUITY UPLC® BEH C18 column (2.1 × 100 mm, 1.8 µm; Waters Corporation, Milford, MA, USA); Dichloromethane (Beijing Tongguang Fine Chemical Company); Nitric oxide (NO) assay kit (Nanjing Jiancheng Bioengineering Institute, Nanjing, China); ELISA kits for Somatostatin (SS), Motilin (MTL), Gastrin (GAS), and Vasoactive intestinal peptide (VIP) (Shanghai Mlbio Biotechnology Co., Ltd.,Shanghai, China); Multi-factor detection kit (RayBiotech (Guangzhou) Biotechnology Co., Ltd., Guangzhou, China); Hematoxylin and Eosin (H&E) staining kit (Wuhan Servicebio Technology Co., Ltd., Wuhan, China); Neutral balsam, absolute ethanol, xylene (Sinopharm Chemical Reagent Co., Ltd.,Beijing, China); Magnetic Soil and Fecal Genomic DNA Extraction Kit and Bacterial Genomic DNA Extraction Kit (Tiangen Biorech (Beijing) Co., Ltd., Beijing, China).

The main instruments included: electronic balance (ML104, Mettler Toledo, Shanghai, China); refrigerated centrifuge (TGL-16, XiangYi Instrument, Changsha, China); Epoch multi-functional microplate reader (BioTek, Winooski, VT, USA); Liquid chromatography-tandem mass spectrometry (LC-MS/MS) analysis was performed using an Ultimate 3000 UHPLC system coupled with an LTQ Orbitrap Velos Pro mass spectrometer (Thermo Scientific, Waltham, MA, USA).

#### 2.1.1 Botanical drug authentication and preparation

Ganhai Weikang Capsules (GHWKCs) are composed of eight botanical drugs according to the official formula approved by the China National Medical Products Administration:

*Atractylodes macrocephala* Koidz [Asteraceae; Atractylodis Macrocephalae Rhizoma]; *Glycyrrhiza uralensis* Fisch. ex DC [Fabaceae; Glycyrrhizae Radix et Rhizoma]; *Sepiella maindroni* de Rochebrune [Sepiidae; Sepiae Endoconcha]; *Gynostemma pentaphyllum* (Thunb.) Makino [Cucurbitaceae; Gynostemmatis Herba]; *Corydalis yanhusu*o W.T.Wang ex Z.Y.Su and C.Y.Wu [Papaveraceae; Corydalis Rhizoma]; *Aurantii Fructus Immaturus* [Rutaceae; Aurantii Fructus Immaturus]; *Phellodendron chinense* C.K.Schneid [Rutaceae; Phellodendri Chinensis Cortex] - *Hippophae rhamnoides* L [Elaeagnaceae; Hippophae Fructus].

All botanical drugs were authenticated by Prof. Hai-Yu Zhao at the Institute of Chinese Materia Medica, China Academy of Chinese Medical Sciences, based on morphological and microscopic characteristics according to the *Chinese Pharmacopoeia* (2025 edition). Voucher specimens have been deposited at the Herbarium of the Institute of Chinese Materia Medica, China Academy of Chinese Medical Sciences (voucher numbers: GHWKCs-2024–01 to GHWKCs-2024–08).

The extraction process was as follows: *Sepiae Endoconcha* and *Gynostemmatis Herba* were pulverized into fine powder and mixed uniformly for later use. The remaining six botanical drugs (*Hippophae Fructus, Glycyrrhizae Radix et Rhizoma*, etc.) were decocted three times with water. The first decoction used 12-fold volume of water with 30 min soaking, followed by 2 h of heating; the second used 10-fold volume for 1.5 h; the third used 8-fold volume for 1 h. The three decoctions were combined, filtered, and concentrated under reduced pressure to a thick paste with a relative density of 1.38 (measured at 60 °C). The actual measured drug-to-extract ratio was approximately 3.6:1.

### 2.2 Composition analysis of GHWKCs decoction

Accurately weigh 1.0 g of GHWKCs dried extract and dissolve it in 25 mL of 70% aqueous methanol. The mixture was sonicated at room temperature for 30 min, cooled to room temperature. The weight loss was compensated with 70% aqueous methanol. The extraction was repeated twice, and the combined filtrates were centrifuged at 13,000 rpm for 10 min. The supernatant was filtered through a 0.22 µm microporous membrane for LC-MS analysis.

The chromatographic separation conditions: The column temperature was maintained at 35 °C. The mobile phase consisted of 0.1% formic acid in water (phase A) and acetonitrile (phase B) with a flow rate of 0.3 mL/min and an injection volume of 2 µL. The gradient elution program was as follows: 0∼3 min, 5% B; 3∼35 min, 5%∼55% B; 35∼40 min, 55%∼95% B; 40∼45 min, 95% B; 45∼45.5 min, 95%∼5% B; 45.5∼50 min, 5% B.

The mass spectrometric conditions: The LTQ Orbitrap Velos Pro mass spectrometer was coupled to the UHPLC system through an electrospray ionization (ESI) source, and operated in both positive and negative ion modes simultaneously. The optimized parameters were as follows: sheath gas flow rate 40 mL/min, auxiliary gas flow rate 10 mL/min, capillary temperature 350 °C, and ESI voltage ±3.5 kV, resolution 30,000. MS^2^ experiments were collected in data-dependent acquisition mode, and the top 5 precursor ions with the highest intensity were selected for fragmentation. The chemical constituents of GHWKCs were identified by comparing the quasi-molecular ion peaks, fragment ion information with the literature reports and public databases (HMDB, MassBank).

Among the 63 identified metabolites, only three compounds were confirmed as legal pharmacopoeial markers according to the *Chinese Pharmacopoeia* (2025 edition): synephrine (derived from *Aurantii Fructus Immaturus*), berberine (derived from *Phellodendri Chinensis Cortex*), and isorhamnetin (derived from *Hippophae Fructus*). These marker compounds are highlighted in Supplementary Table S1. All other identified metabolites are reported as ancillary phytochemical constituents that may contribute to the polypharmacological effects of GHWKCs.

### 2.3 Establishment of functional dyspepsia rat model, grouping and intervention

After 1 week of adaptive feeding, the rats were randomly divided into a control group (Con, n = 6) and a model group. The FD model was established by chronic tail-clamp stress combined with irregular feeding for 4 weeks (Ford et al., 2020): Tail-clamp stress: the rat tail was clamped with a hemostat (1 cm from the tail tip) for 30 min per session, twice a day (morning and afternoon) (Sayuk and Gyawali, 2020); Irregular feeding: food was provided on odd days and withheld on even days, with free access to water throughout the modeling period. Two rats were randomly selected from the model group for model validation: the successful FD model was characterized by reduced mental activity, decreased food and water intake, loose stools, and no obvious gross gastrointestinal lesions.

After successful modeling, the FD rats were randomly divided into model group (Mod, n = 6), mosapride positive control group (MOS, 1.35 mg/kg, n = 6), low-dose GHWKCs group (L-GHWK, 324 mg/kg, n = 6), medium-dose GHWKCs group (M-GHWK, 648 mg/kg, n = 6), and high-dose GHWKCs group (H-GHWK, 1296 mg/kg, n = 6). All treatments were administered once daily via oral gavage for 14 consecutive days. Drug doses were calculated based on the human-to-rat body surface area conversion formula (Nair and Jacob, 2016). The recommended clinical dose of GHWKCs for adults is 2.4 g crude drug/day (6 capsules × 0.4 g/capsule), which corresponds to a rat equivalent dose of 240 mg/kg/day (dry extract). The low (324 mg/kg/day), medium (648 mg/kg/day), and high (1,296 mg/kg/day) doses were set at 0.5×, 1×, and 2× the clinical equivalent dose, respectively, to investigate dose-dependent therapeutic effects. Notably, the high dose exceeds the commonly recommended upper limit of 1 g/kg/day for rodent pharmacological studies and was included specifically to explore the full dose-response relationship.

### 2.4 Specimen collection

After the final administration, all rats were fasted for 12 h with free access to water. Each animal then gavaged with 3 mL of nutrient semi-solid paste containing carbon powder. After 30 min, anesthesia was induced by intraperitoneal injection of 1% sodium pentobarbital (40 mg/kg). Abdominal aortic blood was collected and centrifuged at 3,000 rpm for 10 min at 4 °C to obtain serum, stored at −80 °C. Gastric emptying rate and intestinal propulsion rate were calculated follows:

$$Gastric\;\;emptying\;\;rate=\frac{1-\left(total\;stomach\;weight-empty\;\;stomach\;\;weight\right)}{weight\;of\;semi-solid\;paste\;}\times 100\%$$

$$Intestinal\;propulsion\;\;rate=\frac{distance\;\;\;of\;\;semi-solid\;\;paste\;\;propulsion\;\;in\;\;the\;\;small\;\;intestine\;}{total\;\;length\;\;of\;small\;\;intestine\;}\times 100\%$$

Cecal contents were collected for 16S rRNA sequencing. Gastric and duodenal tissues were collected: fixed in 4% paraformaldehyde for hematoxylin-eosin (H&E) staining or stored at −80 °C for inflammatory factor detection and metabolomics analysis.

### 2.5 Tissues pathological analysis and inflammatory factors detection

The gastric and duodenal tissues fixed in 4% paraformaldehyde were dehydrated with gradient ethanol, cleared with xylene, embedded in paraffin, and cut into 5 µm thick sections. The sections were stained with H&E according to the kit instructions, and sealed with neutral balsam. For cytokine analysis, 100 mg of gastric and duodenal tissues were weighed and homogenized with 900 µL of normal saline on ice. The homogenate was centrifuged at 12,000 rpm for 15 min at 4 °C, and the supernatant was collected. The levels of Interferon-γ (IFN-γ), Interleukin (IL)-6, IL-2, IL-17, IL-4, and IL-10 were quantified with a RayPlex® Rat Inflammation Detection Kit according to the manufacturer’s protocol.

### 2.6 Serum biochemical and gastrointestinal hormone analysis

The concentration of nitric oxide (NO) in serum was determined using a commercially available NO assay kit, in strict accordance with the manufacturer’s instructions. The serum concentrations of the gastrointestinal hormones motilin (MTL), vasoactive intestinal peptide (VIP), gastrin (GAS), and somatostatin (SS) were measured using corresponding enzyme-linked immunosorbent assay (ELISA) kits, following the protocols provided by the manufacturer.

### 2.7 Duodenal metabolomics analysis

#### 2.7.1 Sample processing

FD rats’ duodenum tissues were homogenized into powder on dry ice. 50 mg of powder was weighed and homogenized with 3-fold volume of normal saline for 2 min. Subsequently, 3-fold volume of acetonitrile was added and homogenized for another 2 min. The homogenate was sonicated for 10 min on ice and centrifuged at 12,000 rpm for 15 min at 4 °C. The supernatant was collected for analysis. A quality control (QC) sample was prepared by mixing equal volumes of supernatant from all duodenal samples to evaluate the stability and repeatability of the detection system.

#### 2.7.2 Chromatographic and mass spectrometry conditions

The chromatographic conditions were as follows: The column temperature was maintained at 35 °C; the mobile phase consisting of acetonitrile (phase A) and 0.1% formic acid in water (phase B); flow rate 0.3 mL/min; injection volume 8 µL. The gradient elution program was: 0∼2 min, 5%∼55% A; 2∼10 min, 55%∼95% A; 10∼15 min, 95% A; 15∼15.5 min, 95%∼5% A; 15.5∼20 min, 5% A. The mass spectrometric conditions were consistent with those described in Section 2.2.

#### 2.7.3 Metabolomics data processing and analysis

Raw metabolomics data were collected by Xcalibur 3.0 software (Thermo Fisher Scientific, USA) and processed by Compound Discoverer 3.3 software for peak alignment, peak extraction, and metabolite identification. Quality control (QC) samples were inserted every six injections to monitor system stability, and the relative standard deviation (RSD) of peak areas for all metabolites in QC samples was <15%. Peak areas were normalized to total ion current (TIC) to correct for instrumental fluctuations. Missing values were imputed using the k-nearest neighbor (k-NN) method (k = 5) for metabolites missing in <50% of samples; metabolites missing in >50% of samples were excluded.

Total ion current (TIC) normalization combined with probabilistic quotient normalization (PQN) was applied to eliminate variations in injection volume and dilution effects. For missing value handling, metabolic features with >50% missing values across all samples were excluded, and remaining missing values were imputed using the half-of-the-minimum positive value method.

Differential metabolites were screened using the following criteria (Ford et al., 2020): variable importance in projection (VIP) score >1 from partial least squares discriminant analysis (PLS-DA) (Sayuk and Gyawali, 2020); fold change (FC) > 2 or <0.5; and (Xiao et al., 2021) *P* < 0.05 (Student’s t-test) followed by Benjamini–Hochberg FDR correction (FDR-adjusted *P* < 0.05). Metabolites were identified by matching with the Human Metabolome Database (HMDB) at Level 2 confidence (MS/MS spectral library matching), with the following criteria: mzCloud MS/MS spectral match score >80, exact mass error <10 ppm, and retention time consistent with database reference values. KEGG pathway enrichment analysis of differential metabolites was performed using MetaboAnalyst 6.0 software.

### 2.8 16S rRNA sequencing of intestinal contents and gut microbiota analysis

#### 2.8.1 DNA extraction and PCR amplification

Total genomic DNA was extracted from cecal contents using the Fecal Genomic DNA Extraction Kit according to the manufacturer’s instructions. The V3-V4 hypervariable region of the bacterial 16S rRNA gene was amplified with primers 341F (5′-CCTACGGGNGGCWGCAG-3′) and 805R (5′-GACTACHVGGGTATCTAATCC-3′). The PCR amplification conditions were: pre-denaturation at 98 °C for 30 s; 32 cycles of denaturation at 98 °C for 10 s, annealing at 54 °C for 30 s, extension at 72 °C for 45 s; and final extension at 72 °C for 10 min.

#### 2.8.2 Library construction and sequencing

The PCR products were purified with AMPure XT Beads (Beckman Coulter, Brea, CA, USA), and the concentration was quantified by Qubit 4.0 fluorometer (Thermo Scientific). The library quality was evaluated by Agilent 2100 Bioanalyzer (Agilent Technologies, Santa Clara, CA, USA). The qualified libraries were sequenced on the Illumina NovaSeq 6000 platform (PE250) by LC-Bio Technology Co., Ltd (Hangzhou, China).

#### 2.8.3 Bioinformatics analysis

The raw sequencing data were processed by DADA2 software for quality control, denoising, and ASVs generation. Species annotation of ASVs was performed using QIIME2 software with the SILVA and NT-16S databases (97% similarity). The α-diversity (Chao1, observed species, Shannon index) was calculated to evaluate the microbial richness and diversity, and the β-diversity (PCA) was used to analyze the structural differences of gut microbiota among groups. The relative abundance of gut microbiota at the phylum and genus levels was calculated, and the Linear Discriminant Analysis Effect Size (LEfSe) was used to identify the characteristic flora of each group (LDA score ≥1.9).

### 2.9 Network pharmacology analysis

#### 2.9.1 Active metabolite screening and target prediction

Based on the 63 chemical metabolites of GHWKCs identified in Section 2.2, their SMILES structures were retrieved from the PubChem database. The Swiss ADME platform was used to screen metabolites with favorable gastrointestinal absorption and oral bioavailability (Daina et al., 2017). Potential protein targets were predicted using Swiss Target Prediction with a probability threshold >0.1 (Daina et al., 2019). Detailed ADME parameters of the screened active metabolites are provided in Supplementary Table S2.

#### 2.9.2 Acquisition of FD-related disease targets

Functional dyspepsia (FD)-related targets were collected from Gene Cards (score ≥ median 3.81), the Therapeutic Target Database (TTD), and Online Mendelian Inheritance in Man (OMIM) (Hu et al., 2025; Zhou et al., 2024). After deduplication, intersection targets between GHWKCs active metabolites and FD were obtained using an online Venn diagram tool (Liu et al., 2022).

#### 2.9.3 PPI network construction and core target screening

The intersection targets were uploaded to the STRING database (species: *Homo sapiens*, confidence score: 0.4) to construct a protein–protein interaction (PPI) network (Szklarczyk et al., 2021). The network was visualized and analyzed in Cytoscape 3.9.1 (Shannon et al., 2003). The CentiScape 2.2 plugin was used to calculate degree, betweenness, and closeness centrality. Core targets were defined as those with all three parameters above the median.

#### 2.9.4 GO and KEGG pathway enrichment analysis

Gene Ontology (GO) and Kyoto Encyclopedia of Genes and Genomes (KEGG) enrichment analyses were performed using the DAVID database (Huang da et al., 2007). Terms with *p* < 0.05 were considered statistically significant. The top 10 GO terms and top 20 KEGG pathways were visualized (Kanehisa et al., 2023).

All raw data generated from network pharmacology analysis are provided in the Supplementary Material.

### 2.10 Correlation analysis of differential metabolites and gut microbiota

Spearman correlation analysis was performed to explore the association between the 18 identified differential metabolites and the differential gut microbiota (LDA score ≥1.9) using SPSS 26.0 software. The correlation heatmap was drawn with R 4.2.1 software, and the correlation was considered significant when *p* < 0.05.

### 2.11 Statistical analysis

All data were expressed as mean ± standard error of the mean (SEM) from at least six independent experiments. Statistical analysis was performed using GraphPad Prism 9.5.1 software (GraphPad Software, San Diego, CA, USA). Differences among multiple groups were compared by one-way analysis of variance (ANOVA) followed by Dunnett’s *post hoc* test for comparisons of each treatment group to the model group. Comparisons between two groups were performed using two-tailed Student’s t-test. For multi-omics datasets (16S rRNA sequencing) and LEfSe analyses, the Benjamini–Hochberg false discovery rate (FDR) correction was applied to adjust for multiple comparisons. A P-value <0.05 (or FDR-adjusted P-value <0.05 for multi-omics data) was considered statistically significant.

## 3 Results

### 3.1 Identification of the chemical constituents of GHWKCs

**FIGURE 1:**
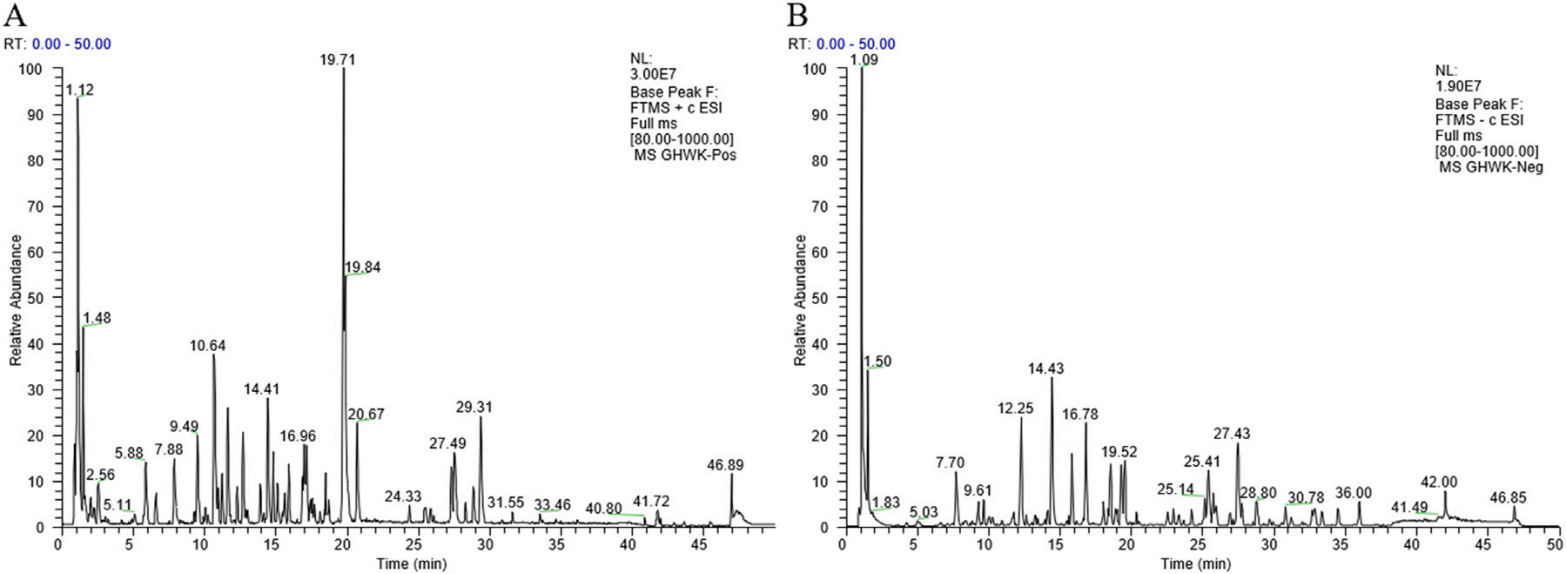
Base peak chromatogram of GHWKCs by UPLC-LTQ-Orbitrap-MS. **(A)** Positive ion mode. **(B)** Negative ion mode.

A total of 63 chemical constituents were identified in GHWKCs by UPLC-LTQ-Orbitrap-MS in positive and negative ion modes, including 31 flavonoids, 10 phenolic compounds, 17 alkaloids, and 5 other metabolites. The base peak chromatograms (BPCs) of GHWKCs in positive and negative ion modes are shown in Figure 1, and the detailed mass spectrometric data of the identified constituents are summarized in Supplementary Table S1. The main active metablolites included baicalin, glycyrrhizic acid, berberine, corydaline, and gypenoside, which are reported to have anti-inflammatory, gastroprotective, and gastrointestinal motility-regulating effects.

### 3.2 GHWKCs improve body weight gain and gastrointestinal motility in FD rats

**FIGURE 2:**
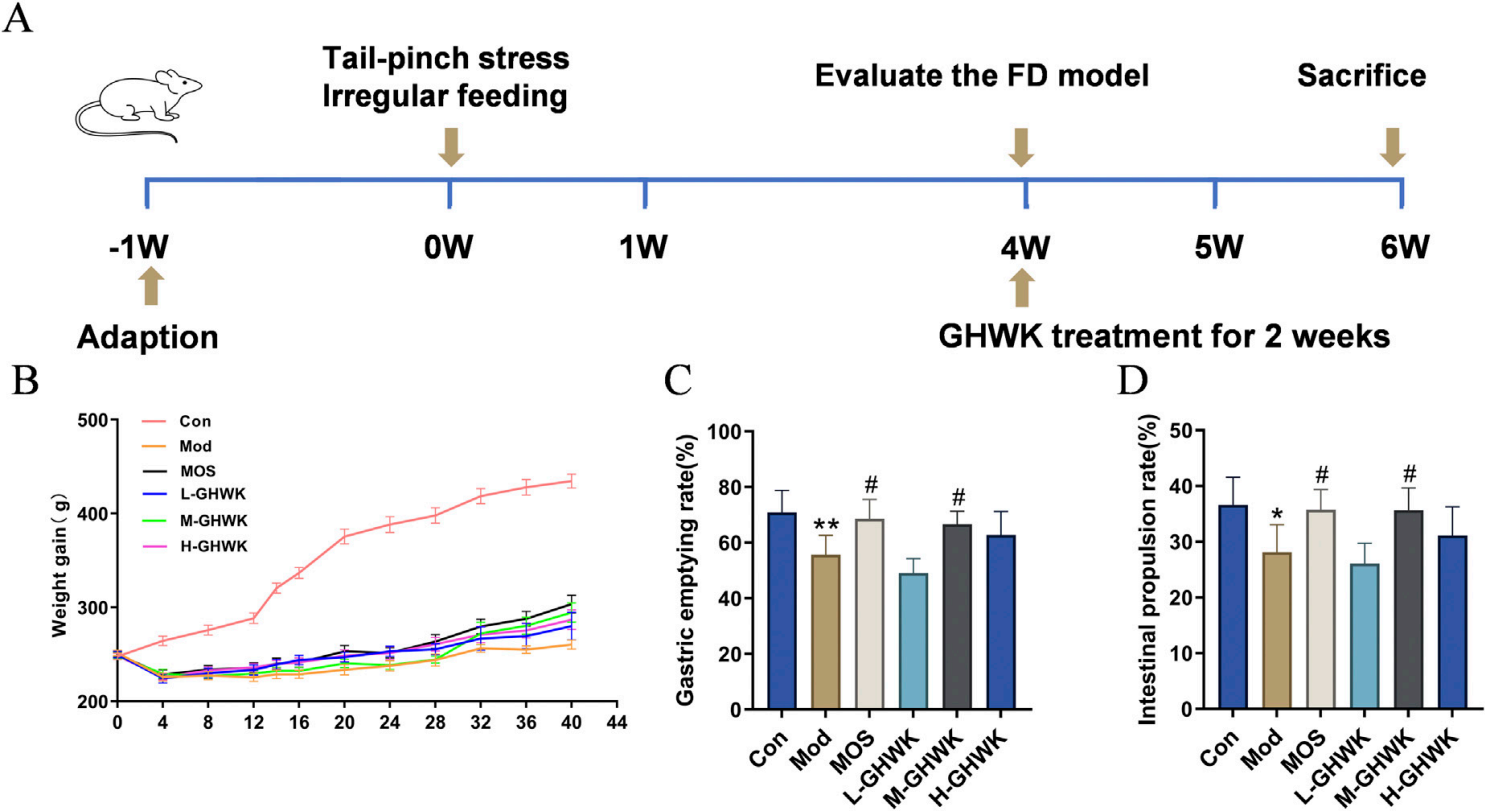
Effects of GHWKCs on body weight and gastrointestinal motility in FD rats. **(A)** Experimental timeline. **(B)** Body weight changes. **(C)** Gastric emptying rate. **(D)** Intestinal propulsion rate. *p < 0.05, **p < 0.01 vs. Con; #*p* < 0.05, ##*p* < 0.01 vs. Mod (n = 6).

The experimental timeline is shown in Figure 2A. During the modeling period, the model group rats showed reduced mental activity, decreased food and water intake, huddling behavior, yellow and withered coat, and loose foul-smelling stools, which were significantly alleviated after GHWKCs or mosapride intervention. As shown in Figure 2B, the body weight gain of the model group was significantly lower than that of the control group (*p* < 0.01), confirming the successful establishment of the FD model. Body weight gain was significantly increased in the mosapride group and the medium/high-dose GHWKCs groups compared with the model group (*p* < 0.05), while the low-dose GHWKCs group showed a slight increase without statistical significance. The Gastrointestinal motility detection results of the gastric emptying rate and intestinal propulsion rate are shown in Figures 2C,D, the model group were significantly lower than those of the control group (both *p* < 0.01). Compared with the model group, the mosapride group significantly improved both indices (*p* < 0.01); the medium/high-dose GHWKCs groups also significantly increased the gastric emptying rate and intestinal propulsion rate (*p* < 0.05), while the low-dose GHWKCs group showed a trend of improvement without statistical significance.

### 3.3 GHWKCs alleviate gastrointestinal mucosal inflammation in FD rats

**FIGURE 3:**
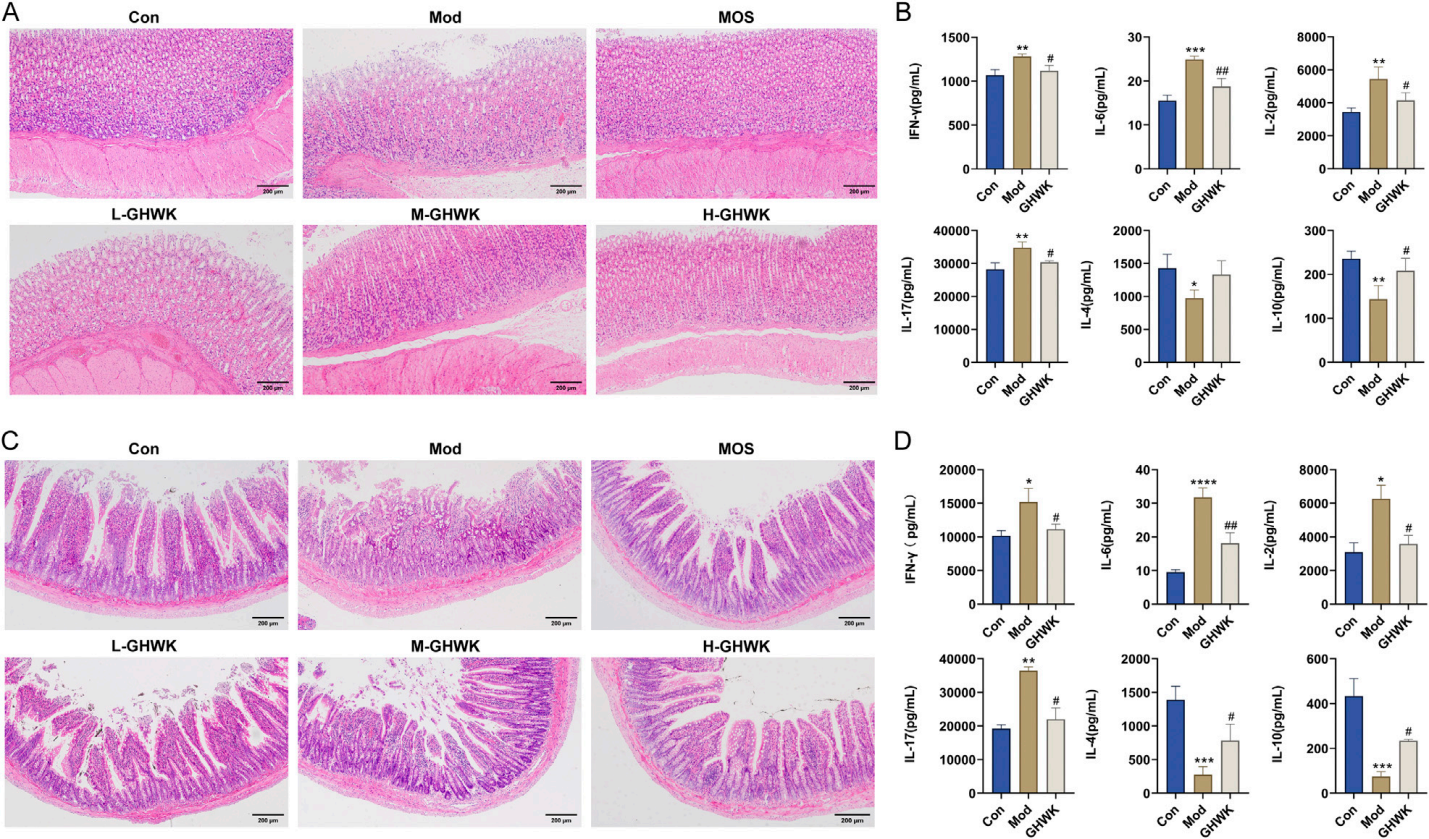
Effects of GHWKCs on gastrointestinal mucosal histopathology and inflammatory factors in FD rats. **(A)** Gastric tissue H&E staining (scale bar: 200 μm); **(B)** Gastric tissue inflammatory factors; **(C)** Duodenal tissue H&E staining (scale bar: 200 μm). **(D)** Duodenal tissue inflammatory factor. *p < 0.05, **p < 0.01, ***p < 0.001 vs. Con; #*p* < 0.05, ##*p* < 0.01 vs. Mod (n = 6).

Gastrointestinal tissue pathological changes in FD rats were examined by hematoxylin-eosin (H&E) staining. Gastric tissue results were shown in Figure 3A, the control group (Con) presented an intact morphological structure with neatly arranged epithelial cells and no visible damage whereas the model group (Mod) exhibited disordered gastric gland arrangement, widened epithelial intercellular spacing, inflammatory cell infiltration in the lamina propria, and obvious submucosal edema. Compared with the model group, the mosapride group (MOS), medium-dose (M-GHWK) and high-dose (H-GHWK) GHWKCs groups essentially restored gastric morphological structure, reduced inflammatory cell numbers, alleviated submucosal edema, with only a small amount of residual epithelial cell shedding observed. Duodenal tissue results are shown in Figure 3C, the control group had a complete tissue structure with clear morphology and neatly arranged goblet cells without obvious lesions, while the model group showed blunted, significantly shortened or atrophic intestinal villi, disordered goblet cell arrangement, and inflammatory cell infiltration in the intestinal wall. Intervention with mosapride, medium or high-dose GHWKCs improved intestinal villus alignment and reduced intestinal wall inflammatory cell infiltration in comparison with the model group. The levels of pro-inflammatory factors (interferon-γ [IFN-γ], interleukin-6 [IL-6], IL-2, IL-17) and anti-inflammatory factors (IL-4, IL-10) in gastrointestinal tissues were detected by ELISA. Gastric tissues results were shown in Figure 3B, the model group showed a significant elevation in IFN-γ, IL-6, IL-2, and IL-17 expression, and a marked reduction in IL-4 and IL-10 expression compared with the control group (*p* < 0.05, *p* < 0.01, *p* < 0.001). Compared with the model group, GHWKCs treatment significantly downregulated the expression of the four pro-inflammatory cytokines (*p* < 0.01) and upregulated IL-10 levels (*p* < 0.05). Duodenal tissues results were shown in Figure 3D, the results were consistent with those of gastric tissues: GHWKCs-treated groups exhibited a significant reduction in pro-inflammatory factors and an increase in the anti-inflammatory factor IL-10 compared with the model group. Notably, GHWKCs also significantly upregulated IL-4 expression in duodenal tissues (*p* < 0.05).

### 3.4 GHWKCs regulate the balance of serum gastrointestinal hormone and NO levels in FD rats

**FIGURE 4:**
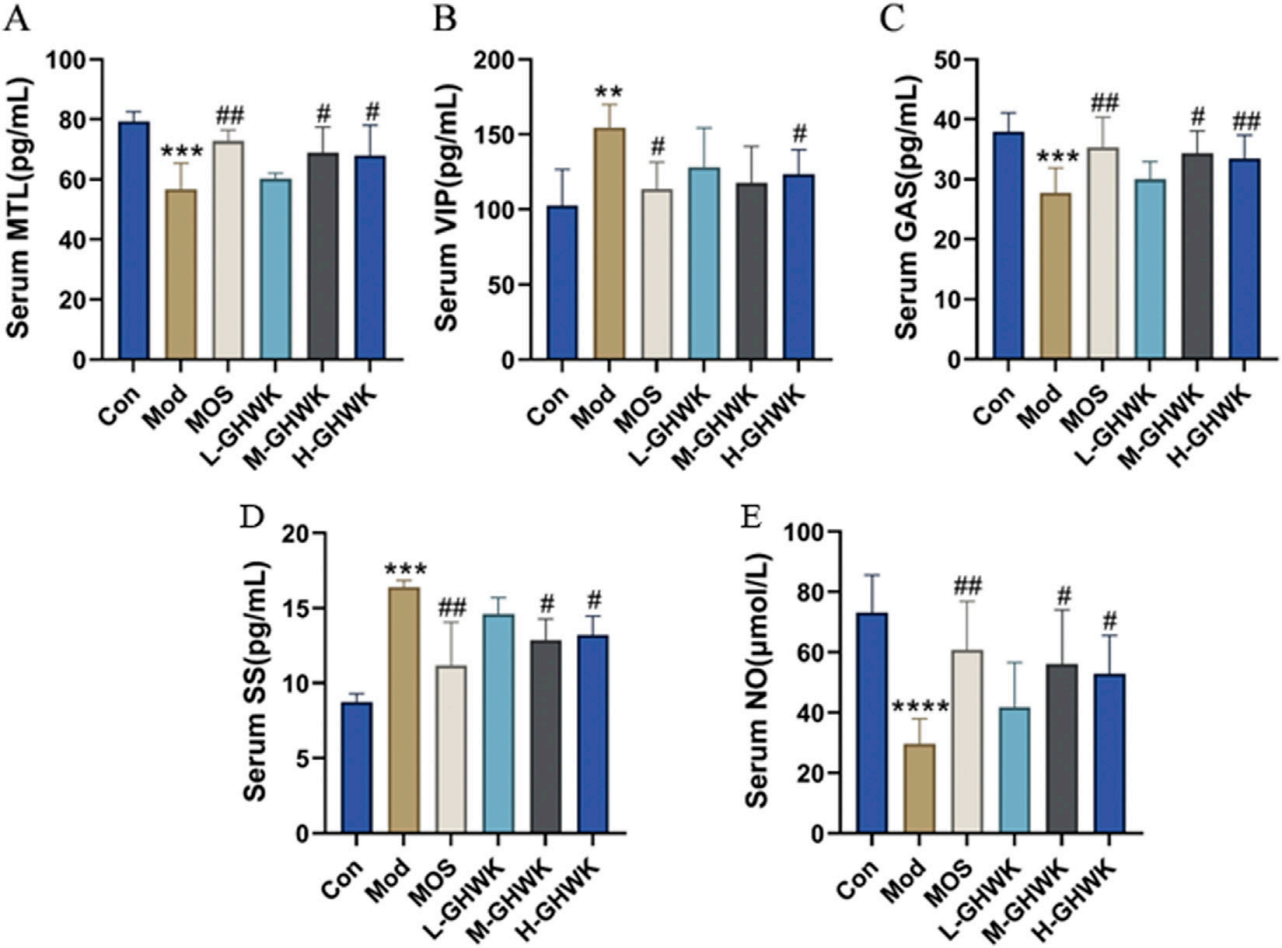
Effects of GHWKCs on serum gastrointestinal hormones and NO levels in FD rats **(A)** Serum motilin (MTL), **(B)** Serum vasoactive intestinal peptide (VIP), **(C)** Serum gastrin (GAS) **(D)** Serum somatostatin (SS), **(E)** nitric oxide (NO). *p < 0.05, **p < 0.01, ***p < 0.001 vs. Con; #*p* < 0.05, ##*p* < 0.01 vs. Mod (n = 6).

Serum samples were collected from rats after the final drug administration. The concentrations of gastrointestinal hormones, including motilin (MTL), gastrin (GAS), vasoactive intestinal peptide (VIP), and somatostatin (SS), as well as nitric oxide (NO), were measured using commercially available ELISA kits and an NO detection kit, following the manufacturers’ protocols. The results are shown in Figure 4. Compared with the control group, the model group had significantly decreased serum levels of MTL, GAS, and NO (*p* < 0.001), alongside significantly increased levels of VIP and SS (*p* < 0.01). Treatment with mosapride significantly increased the serum concentrations of MTL, GAS, and NO (*p* < 0.01), and decreasing the levels of VIP (*p* < 0.05) and SS (*p* < 0.01) relative to the model group. Both medium and high-doses of GHWKCs significantly upregulated the serum levels of MTL, GAS, and NO (*p* < 0.05); The medium-dose of GHWKCs group also significantly reduced VIP and SS levels (*p* < 0.05), and the high-dose GHWKCs group significantly decreased SS levels (*p* < 0.05). The low-dose GHWKCs group showed a trend of regulating these indices without statistical significance.

### 3.5 GHWKCs restore duodenal metabolic homeostasis in FD rats

**FIGURE 5:**
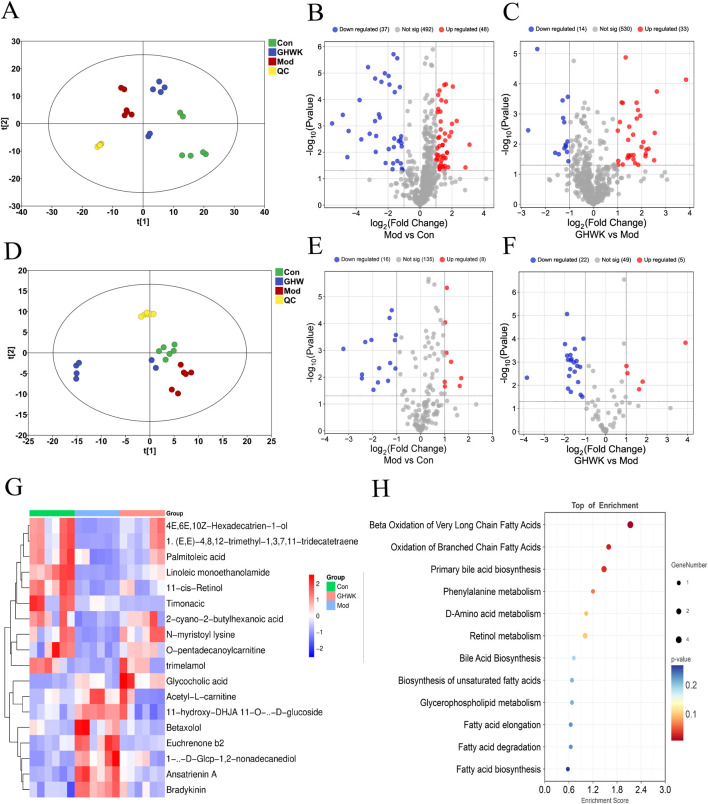
Duodenal metabolomics analysis of FD rats after GHWKCs intervention. **(A)** PCA score plot (positive ion mode); **(B)** Volcano plot (Mod vs. Con, positive); **(C)** Volcano plot (GHWK vs. Mod, positive); **(D)** PCA score plot (negative ion mode); **(E)** Volcano plot (Mod vs. Con, negative); **(F)** Volcano plot (GHWK vs. Mod, negative); **(G)** Heatmap of 18 differential metabolites; **(H)** KEGG pathways enrichment bubble plot.

Based on duodenal metabolomics analysis using ultra-performance liquid chromatography-linear trap quadrupole-Orbitrap mass spectrometry (UPLC-LTQ-Orbitrap-MS), a total of 736 metabolites were identified, the analysis results are shown in Figure 5. Among them, Figures 5A,D present the principal component analysis (PCA) results, which reveal a clear separation between the control and model groups, indicating that the modeling procedure induced significant metabolic perturbations. Notably, the GHWKCs group was distinctly separated from the model group in the clustering analysis, suggesting a restorative effect on metabolic homeostasis. The tight clustering of quality control (QC) samples in the PCA score plot validated the stability and reliability of the analytical sequence. Using screening criteria of fold change (FC) > 2 or <0.5, P-value <0.05, and variable importance in projection (VIP score) > 1, 85 metabolites were identified as significantly altered in the model group compared to the control group. Specifically, Figure 5B shows that in the positive ion mode, 48 metabolites were upregulated and 37 were downregulated, while Figure 5E shows that in the negative ion mode, 33 were upregulated and 14 were downregulated. Compared to the model group, the GHWKCs group exhibited 24 differential metabolites in the positive ion mode (8 upregulated and 16 downregulated), as shown in Figure 5C, and 60 differential metabolites in the negative ion mode (5 upregulated and 22 downregulated), as shown in Figure 5F. Subsequently, 18 metabolites were unambiguously identified by matching with the Human Metabolome Database (HMDB) and are listed in Table 1. Figure 5G displays a heatmap of the relative abundances of these metabolites across different groups. The 18 identified differential metabolites in Table 1 were further subjected to pathway enrichment analysis using Metabo Analyst 6.0. The results were shown in Figure 5H, it revealed significant enrichment in multiple metabolic pathways, including β-oxidation of very long-chain fatty acids, oxidation of branched-chain fatty acids, and primary bile acid biosynthesis. Given that disordered bile acid metabolism is known to impair the intestinal mucosal barrier and is implicated in the pathogenesis of functional dyspepsia (FD) (Gu et al., 2022), the primary bile acid biosynthesis pathway was highlighted as a potentially crucial pathway mediating the therapeutic effect of GHWKCs.

**TABLE 1:** Identification of differential metabolites in the duodenum of FD rats after GHWKCs intervention.

No.	Metabolite	Formula	*m/z*	rt(min)	Mode	Mod vs. Con	GHWKCs vs. Mod
1	11-cis-Retinol	C_20_H_30_O	269.225	6.039	[M + H] ^-^	↓***	↑*
2	1-O-alpha-D-glucopyranosyl-1,2-nonadecandiol	C_25_H_50_O_7_	463.363	5.099	[M + H] ^+1^	↑***	↓*
3	2,6,10-Trimethyl-(E, E)-2,6,10,12-tridecatetraene	C_16_H_26_	219.210	10.260	[M + H] ^+1^	↓***	↑*
4	2-Cyano-2-butylhexanoic acid	C_11_H_19_NO_2_	198.148	11.774	[M + H] ^+1^	↓**	↑**
5	4E,6E,10Z-Hexadecatrien-1-ol	C_16_H_28_O	237.220	10.266	[M + H] ^+1^	↓***	↑*
6	Linoleic monoethanolamide	C_20_H_37_NO_2_	324.289	5.309	[M + H] ^+1^	↓***	↑**
7	Ansatrienin A	C_36_H_48_N_2_O_8_	637.353	2.971	[M + H] ^+1^	↑***	↓***
8	Betaxolol	C_18_H_29_NO_3_	308.221	3.396	[M + H] ^+1^	↑*	↓*
9	Euchrenone b2	C_30_H_34_O_6_	491.242	1.235	[M + H] ^+1^	↑**	↓**
10	Glycocholic acid	C_26_H_43_NO_6_	466.315	3.530	[M + H] ^+1^	↓*	↑*
11	N-myristoyl lysine	C_20_H_40_N_2_O_3_	357.310	4.261	[M + H] ^+1^	↓**	↑***
12	O-pentadecanoylcarnitine	C_22_H_43_NO_4_	386.325	5.343	[M + H] ^+1^	↓*	↑***
13	Palmitoleic acid	C_16_H_30_O_2_	253.217	10.261	[M-H] ^−1^	↓**	↑**
14	Bradykinin	C_23_H_35_N_7_O_6_	506.269	3.040	[M + H] ^+1^	↑***	↓***
15	Timonacic	C_4_H_7_NO_2_S	134.027	1.055	[M + H] ^+1^	↓*	↓**
16	Trimelamol	C_9_H_18_N_6_O_3_	259.152	1.489	[M + H] ^+1^	↓**	↑*
17	Acetyl-L-carnitine	C_9_H_17_NO_4_	202.107	3.172	[M-H] ^−1^	↑*	↓***
18	(−)-11-Hydroxy-9,10-dihydrojasmonic acid 11-beta-D-glucoside	C_18_H_30_O_9_	389.183	2.802	[M-H] ^−1^	↑***	↓**

*p < 0.05; **p < 0.01; ***p < 0.001; ↓: downregulated; ↑: upregulated.

### 3.6 GHWKCs reshape the gut microbiota structure in FD rats

**FIGURE 6:**
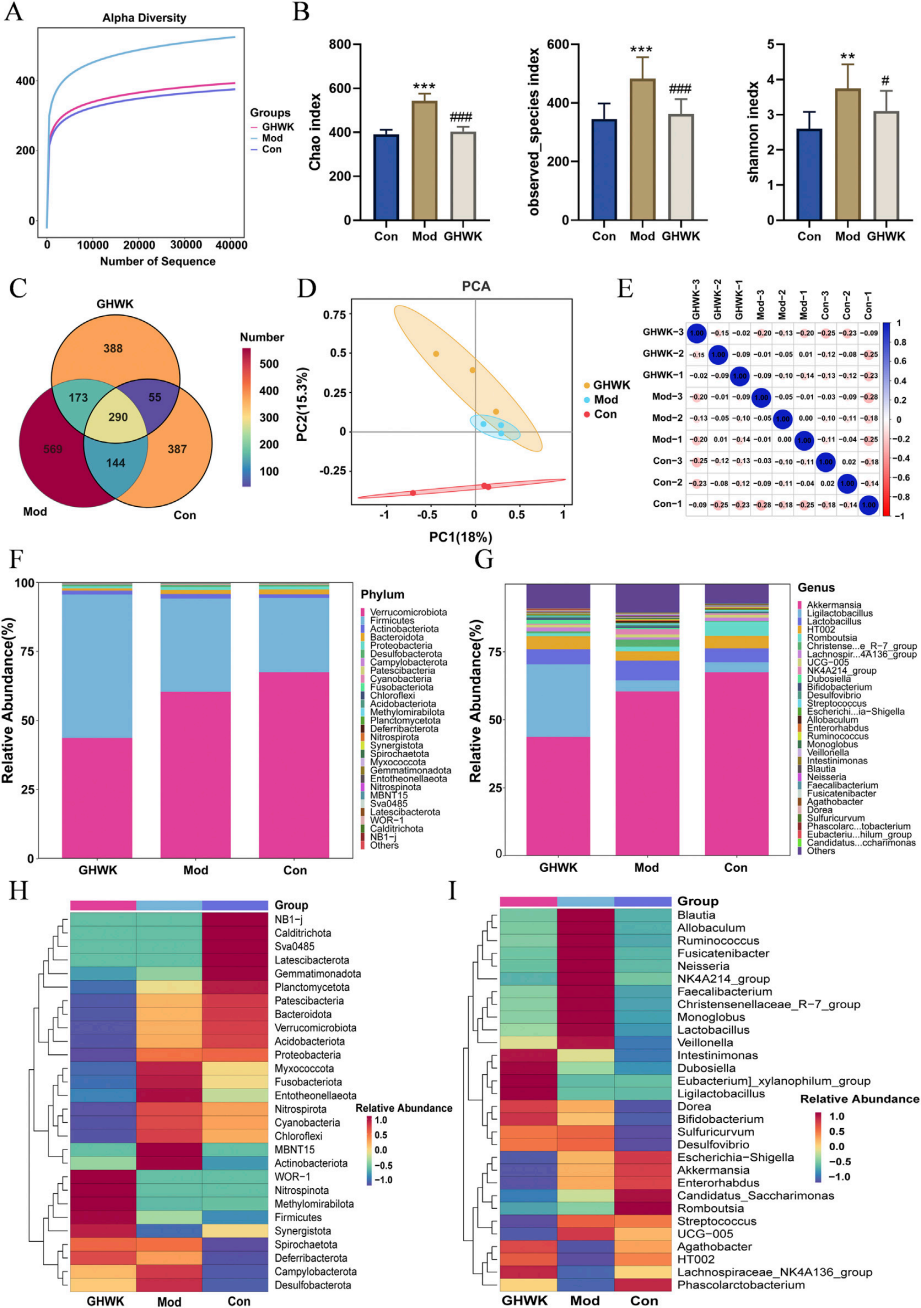
Gut microbiota analysis in FD rats after GHWKCs intervention **(A)** Rarefaction curves **(B)** α-diversity indices; **(C)** Venn diagram of ASVs; **(D)** PCA of β-diversity; **(E)** Samples correlation heatmap. **(F)** Top 20 phyla relative abundance; **(G)** Phylum-level heatmap; **(H)** Top 20 genera relative abundance. **(I)** Genus-level taxa heatmap. *p < 0.05, **p < 0.001 vs. Mod (n = 6).

Given the implicated role of gut microbiota in FD, we systematically characterized the gut microbial community structure in an FD rat model, with the results presented in Figure 6. First, we assessed microbial diversity. As shown in Figure 6A, the rarefaction curves plateaued, confirming sufficient sequencing depth. Figure 6B presents the α-diversity analysis as evaluated using the Chao1 index, observed species count, and Shannon index. The α-diversity analysis revealed that all metrics (Chao1 index, observed species count, and Shannon index) were significantly higher in the model group compared with the control group, indicating microbial dysbiosis. Treatment with GHWKCs significantly decreased these indices toward normal levels (*p < 0.05; **p < 0.001 vs. Mod), suggesting a restorative effect on gut microbial ecology. Figure 6C presents the Venn diagram showing the numbers of effective Amplicon Sequence Variants (ASVs) in the gut microbiota of rats across three groups: 876 in the normal control group, 1176 in the model group, and 906 in the GHWKCs group. The ASVs of the three groups had both overlaps and each possessed unique taxa, indicating that the microbial community structure of the model group might have undergone changes, and GHWKCs could partially restore the intestinal microecological composition of model rats. Figure 6D displays the β-diversity analysis based on principal component analysis (PCA), demonstrating clear and distinct separation among microbial communities across experimental groups. Next, a comparative analysis of changes in the rat gut microbiota was performed. Figures 6F,G show that at the phylum-level, the gut microbiota of FD rats was predominantly composed of *Verrucomicrobiota, Firmicutes, Actinobacteriota, Bacteroidota,* and *Proteobacteria*. Compared with the control group, the model group exhibited a significant decrease in the relative abundances of *Verrucomicrobiota, Actinobacteriota,* and *Bacteroidota,* whereas *Firmicutes* and *Proteobacteria* were significantly enriched. Figures 6H,I present the genus-level analysis, revealing that high-abundance genera such as *Akkermansia*, *Ligilactobacillus*, *Lactobacillus*, and HT002 in the model group were significantly reduced, whereas *Romboutsia* was markedly enriched. Notably, intervention with GHWKCs effectively reversed these dysbiosis alterations, restoring the relative abundances of the aforementioned phyla and genera toward near-normal levels. These findings demonstrate that GHWKCs can markedly reshape the gut microbiota structure in FD rats, driving a robust and beneficial ecological reconfiguration.

### 3.7 Correlation between differential metabolites and gut microbiota in FD rats

**FIGURE 7:**
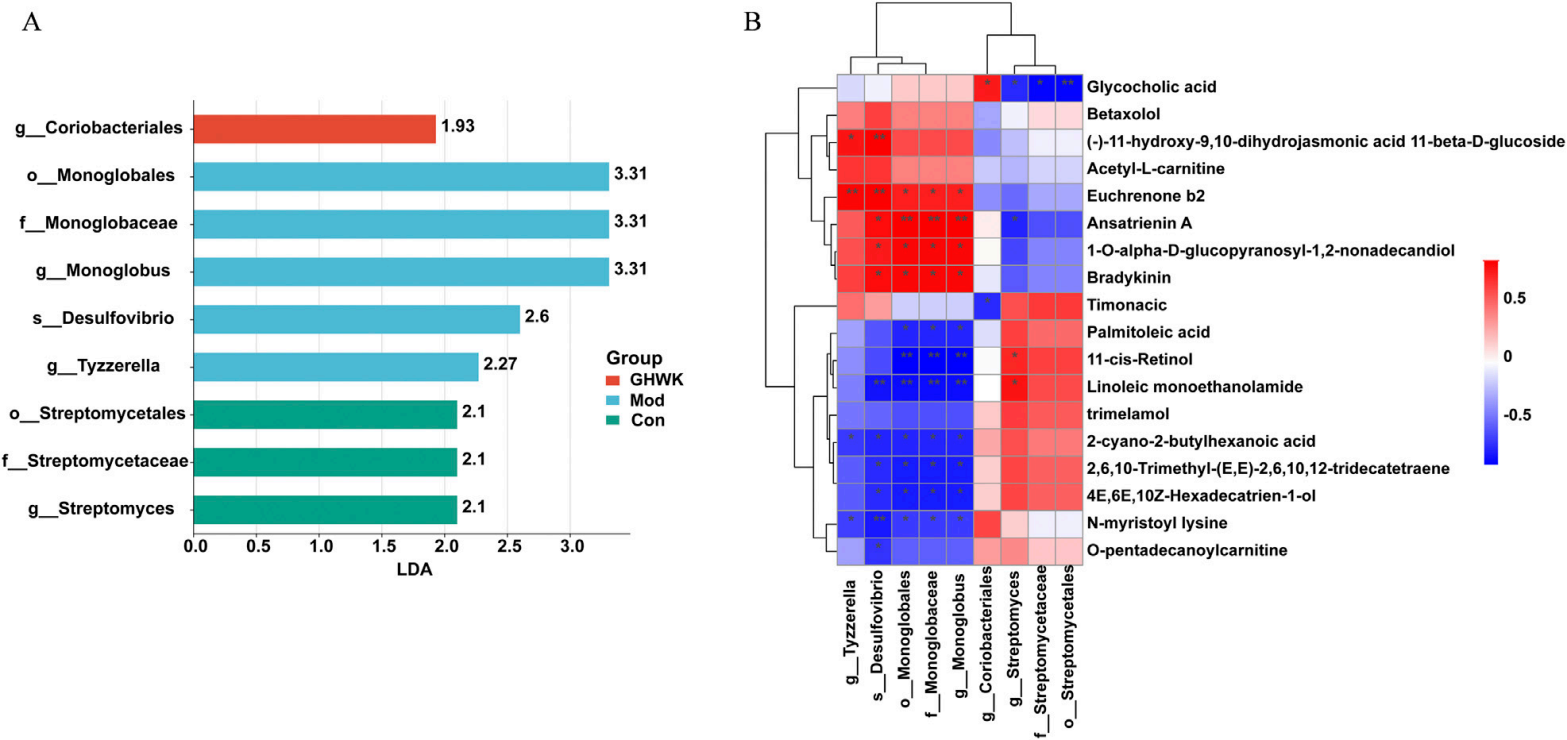
Correlation analysis of differential metabolites and gut microbiota **(A)** LEfSe analysis of characteristic flora (LDA score ≥1.9); **(B)** Spearman correlation heatmap. *p < 0.05, **p < 0.01, ***p < 0.001.

In LEfSe analysis, microorganisms with an LDA scores ≥1.9 in each group were considered to exhibit inter-group differences. The LEfSe analysis results from Figure 7A indicate that: the characteristic flora of the control group were *Streptomycetales*, *Streptomycetaceae*, *Streptomyces*, and *Actinomycetes*; the characteristic genera of the model group were *Monoglobus*, *Desulfovibrio*, and *Tyzzerella*; while the characteristic flora of the GHWKCs group were *Coriobacteriales* and *Actinomycetes*. To further explore potential associations between microbiota and metabolites, Spearman correlation analysis was performed. Figure 7B shows the correlation analysis plot between 18 differential metabolites screened from duodenal metabolomics and 9 differential microbial groups obtained from LEfSe analysis. The results revealed that: in the control group, the differentially enriched *Streptomycetales, Streptomycetaceae,* and *Streptomyces* showed significant positive correlations with multiple differential metabolites, including *Palmitic acid*, *Linoleic acid monoethanolamide*, *2-cyano-2-butylhexanoic acid*, *4E,6E,10Z-hexadecanotrienes-1-alcohol*, and *N-myrisyllysine*; in the GHWKCs group, the differentially enriched *Coriobacteriales* showed a significant positive correlation with *Glycocholic acid*; whereas in the model group, the differentially enriched *Monoglobus, Desulfovibrio,* and *Tyzzerella* showed significant positive correlations with differential metabolites such as euchrenone *B2, 1-O-alpha-D-glucopyranosyl-1,2-nonadecanediol,* and *Bradykinin*, etc., respectively.

### 3.8 Network pharmacology analysis of GHWKCs against FD

#### 3.8.1 Active metabolites and intersection targets

**FIGURE 8:**
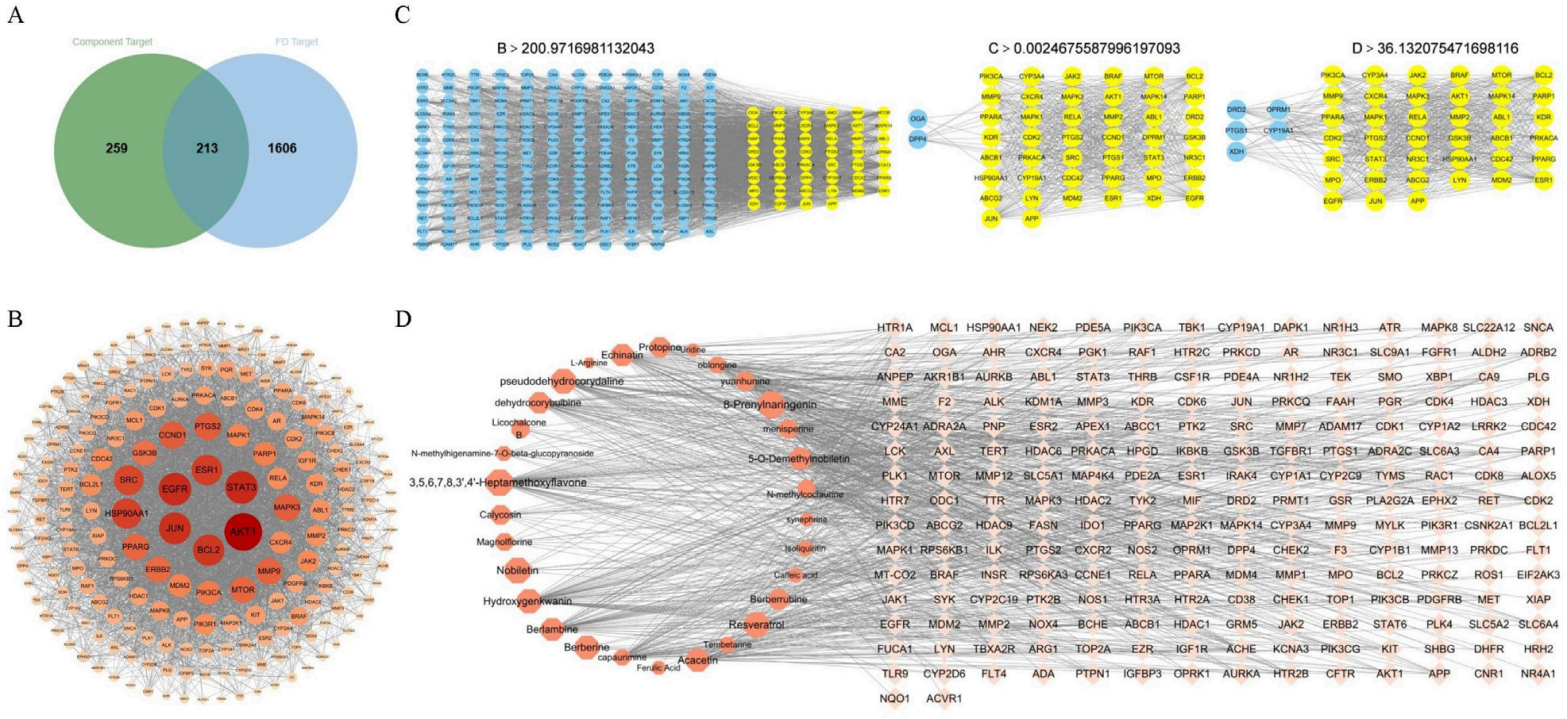
Network pharmacology analysis of Ganhai Weikang Capsules (GHWKCs) against functional dyspepsia (FD). **(A)** Venn diagram showing the intersection of GHWKCs metabolite targets and FD disease targets; **(B)** Protein-protein interaction (PPI) network of the 213 intersection targets **(C)** Topological parameter screening of core targets (core targets defined as those with degree, betweenness, and closeness centrality all above the median) **(D)** “Metabolite-core target” interaction network showing the multi-target regulatory characteristics of GHWKCs.

Thirty-one active metabolites meeting the oral bioavailability and gastrointestinal absorption criteria were screened from 63 identified metabolites (detailed ADME parameters in Supplementary Table S2). These metabolites were predicted to target 472 potential human proteins. After intersecting with 1819 FD-related disease targets, 213 common therapeutic targets were identified, as shown in Figure 8A. The full list in intersection targets is provided in Supplementary Table S3.

#### 3.8.2 PPI network and core target

The PPI network of the 213 intersection targets was constructed, consisting of 213 nodes and 1606 edges, as presented in Figure 8B. Topological analysis based on degree, betweenness, and closeness centrality identified 39 core targets with high connectivity, as shown in Figure 8C. Detailed topological parameters of these core targets are listed in Supplementary Table S4. The top five core targets ranked by degree centrality were ATP-binding cassette subfamily B member 1 (ABCB1), matrix metalloproteinase 2 (MMP2), cyclin D1 (CCND1), nuclear receptor subfamily 3 group C member 1 (NR3C1), and mouse double minute 2 homolog (MDM2). The “metabolite-core target” interaction network was constructed to visualize the multi-target regulatory characteristics of GHWKCs, as presented in Figure 8D (Shannon et al., 2003; Liu et al., 2023).

#### 3.8.3 GO and KEGG enrichment analysis

**FIGURE 9:**
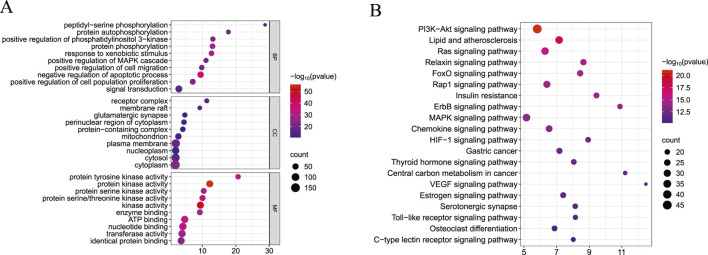
Functional enrichment analysis of the intersection targets. **(A)** Top 10 significantly enriched Gene Ontology (GO) terms (biological process, cellular component, and molecular function) **(B)** Top 20 significantly enriched Kyoto Encyclopedia of Genes and Genomes (KEGG) signaling pathways. The size of the bubble represents the number of enriched genes, and the color represents the P-value.

GO enrichment showed that biological processes were mainly enriched in positive regulation of PI3K signaling, protein phosphorylation, and cell proliferation. Cellular metabolites were concentrated in the plasma membrane and membrane rafts. Molecular functions were dominated by protein kinase activity, as shown in Figure 9A.

KEGG analysis identified 177 significant pathways, with the top 20 including PI3K-Akt, MAPK, chemokine, and Toll-like receptor signaling pathways. These pathways are highly consistent with our *in vivo* experimental findings, as they are closely associated with the gastrointestinal inflammation, mucosal injury, and motility disorders observed in FD rats, as presented in Figure 9B (Kanehisa et al., 2023; Du et al., 2018; Keely and Talley, 2020).

It should be noted that network pharmacology analysis provides predictive hypotheses rather than direct biological validation. The predicted targets and pathways, including NR3C1, ABCB1, PI3K-Akt, and MAPK signaling, are consistent with our *in vivo* observations and provide mechanistic hypotheses that warrant further experimental verification.

## 4 Discussion

FD has a rising prevalence in the context of increasing stress levels in modern society, with core symptoms including postprandial fullness, early satiety, and epigastric pain (Aziz et al., 2018). Pathophysiological studies indicate that delayed gastric emptying is observed in 20%–35% of FD patients (Sarnelli et al., 2003), while approximately 40% of cases exhibit impaired gastric fundic accommodation (Tack et al., 1998). Notably, psychiatric comorbidities are common; depression and anxiety have been reported in 34.36% and 25.55% of FD patients, respectively (Zhang et al., 2016). A dual-factor model was established in rats by combining chronic tail-pinch stress with irregular feeding patterns (Wang et al., 2020). Tail-pinching induces anxiety-depression-like behaviors, while disrupted feeding rhythms trigger gastrointestinal motility disorders. During the modeling period, rats in the model group displayed significant behavioral irritability and increased aggression. Moreover, compared with the control group, the model animals showed reductions in body weight, food intake, and water consumption. Gastric emptying rate and intestinal propulsion rate were also significantly lower, confirming the successful induction of core FD features at both behavioral and physiological levels.

Emerging evidence underscores the significance of low-grade duodenal mucosal inflammation in the pathophysiology of FD (Hari et al., 2022). Multiple studies confirm that FD patients often exhibit compromised duodenal mucosal barrier integrity, leading to increased intestinal permeability and activation of local immune responses (Leites et al., 2024). There is a trend toward increased expression of eosinophils, mast cells, and inflammatory mediators such as IL-1β and TNF-α in the stomach and duodenum, thereby exacerbating local low-grade inflammation (Du et al., 2018). Notably, intestinal glial cells have been found to co-localize with these activated eosinophils and mast cells, an interaction potentially contributing to neuronal sensitization and sustained excitation (Tanaka et al., 2016; Reed et al., 2003). Inflammatory mediators released in this milieu, such as 5-hydroxytryptamine (5-HT) and histamine, can bind to specific receptors widely expressed on primary afferent neurons, thereby playing a critical role in modulating gastrointestinal motility and visceral sensation (Gershon and Tack, 2007; Bueno and Fioramonti, 2002). Histopathology confirmed FD rats displayed gastric gland disarray, inflammatory foci, villous shortening, and epithelial damage, accompanied by dense mucosal and submucosal inflammatory infiltrates and upregulated levels of pro-inflammatory cytokines (e.g., IL-1β, IL-6, TNF-α). GHWKCs significantly improves gastric and duodenal structure while concurrently downregulating these pro-inflammatory factors. This formula contains multiple anti-inflammatory active metabolites, such as atractylenolide III which inhibits IL-1β, IL-6, and TNF-α (Wang et al., 2024), and licorice extract which modulates Bcl-2/Bax to promote repair (Peng et al., 2025). This suggests that the anti-inflammatory and mucosal restorative effects of GHWKCs are achieved through a multi-target mechanism.

In the pathogenesis of FD, dysregulation of the network involving gastrointestinal hormones and signaling molecules plays a critical role (Camilleri, 2019). MTL, a hormone that promotes gastrointestinal motility, can lead to delayed gastric emptying when its secretion is insufficient (Wang et al., 2026). In contrast, SS, a potent inhibitory hormone, can significantly inhibit antral motility and exacerbate motility disorders when overexpressed (Liu et al., 2025). GAS not only stimulates gastric acid secretion and mucosal repair but also plays an important role in maintaining the integrity of the duodenal mucosa (Gao et al., 2025). VIP participates in motility regulation by mediating smooth muscle relaxation, and its’ signaling abnormalities are associated with impaired intestinal barrier function and altered permeability (Bai et al., 2024). Additionally, NO exerts dual regulatory functions: it affects gastrointestinal motility by inducing smooth muscle relaxation as a neurotransmitter and also protects the mucosa by maintaining mucosal blood flow and inhibiting inflammation (Han et al., 2021). The results of this experiment showed that the expression of MTL and GAS in the serum of rats in the model group was significantly reduced, while the levels of VIP and SS were significantly increased. After treatment, the levels of NO, MTL and GAS in the serum of the GHWKCs administration group and the mosapride group increased, while the levels of VIP and SS decreased. GHWKCs may restore normal gastrointestinal motility by reshaping the disrupted gastrointestinal hormone environment through a synergistic mechanism that downregulates inhibitory hormones and upregulates excitatory hormones.

In the non-targeted metabolomics results, the metabolites that changed in the FD model group were mainly concentrated in fatty acids and bile acids, which were adjusted back after intervention with GHWKCs, compared with the control group. Long-chain fatty acids such as palmitic acid directly inhibit gastric motility by activating G protein-coupled receptors on the surface of intestinal endocrine cells and promoting the release of cholecystokinin (Ghezzal et al., 2020). Bile acids are synthesized by the liver and modified by the gut microbiota to secondary bile acids. Bile acids regulate lipid digestion, metabolic homeostasis and immune response through the enterohepatic circulation (Yang et al., 2021). Bile acids promote fat digestion through emulsification, and when metabolism is disrupted, they affect digestion and cause a feeling of fullness after early saturation meals (Song et al., 2025). A change in the ratio of primary bile acids to secondary bile acids may increase the expression of barrier proteins such as JAM-A, ZO-1, and ZO-2, causing damage to the duodenal mucosal barrier, increased permeability, and inducing FD (Keely and Talley, 2020). In addition, studies have shown that the accumulation of bile acids in the gut microbiota further promotes bacterial inhibition by influencing ribose function and amino acid metabolism (Peng et al., 2024). In summary, the gut microbiota modifies the chemical structure of bile acids, which plays a crucial role in intestinal homeostasis by creating the bidirectional regulation of microbiota ecology through antibacterial properties.

In recent years, accumulating evidence has indicates that dysbiosis of the gastrointestinal microbiota contributes significantly to the pathogenesis of FD (Chavez-Arroyo et al., 2025). Under physiological conditions, the gut microbiota maintains a dynamic ecological balance through host-microbe co-metabolism, which supports overall health (Yu et al., 2023). When the abundance and composition of the gastrointestinal microbiota change, the number of beneficial bacteria in the gut decreases while the number of pathogenic bacteria increases (Zhou et al., 2022). Proteobacteria, Firmicutes, Actinobacteria and Bacteroidetes constitute the predominant gastrointestinal microorganisms, collectively representing over 98% of the total gut microbiota (Huang et al., 2019). An increased abundance of Proteobacteria is a sign of dysbiosis in the gastrointestinal microbiota and can be used as a potential diagnostic criterion for disease (Shin et al., 2015). 16S rRNA sequencing results indicated that Firmicutes, Actinomycetes, and Bacteroidetes were the dominant microbiota with higher abundance, and Proteobacteria were the marker microbiota in the LEfSe analysis model group with increased abundance, suggesting that the gastrointestinal microbiota in FD rats may be disordered. In GHWKCs group, Actinomycetes, a marker bacterium, was positively correlated with compounds related to the bile acid metabolic pathway in the metabolome.

Network pharmacology further supported the multi-component, multi-target, and multi-pathway therapeutic properties of GHWKCs for FD. The core target NR3C1 (glucocorticoid receptor) regulates chronic stress and inflammatory responses, which is highly consistent with our chronic tail-clamp stress FD model (Zhang et al., 2016; Wang et al., 2020). ABCB1 acts as a key bile acid transporter in enterohepatic circulation, supporting our metabolomic finding that GHWKCs restore primary bile acid biosynthesis and modulate the *Coriobacteriales*-glycocholic acid axis (Yang et al., 2021; Peng et al., 2024). Moreover, the PI3K-Akt and MAPK pathways are central regulators of duodenal micro-inflammation and mucosal barrier repair (Du et al., 2018; Camilleri, 2019), which strongly supports our *in vivo* observations that GHWKCs inhibit pro-inflammatory cytokines and protect gastric and duodenal mucosa. Together, network pharmacology predictions and experimental findings form a complete mechanistic chain, enhancing the reliability and scientific basis of this study.

From the perspective of TCM theory, the pathogenesis of FD is mainly attributed to spleen deficiency and qi stagnation, which leads to dysfunction of stomach qi descending. The therapeutic principle of GHWKCs is to invigorate the spleen and replenish qi, promote qi circulation, and relieve pain, which is consistent with the TCM treatment strategy for FD. Our modern scientific findings provide molecular evidence for this traditional theory: the improvement of gastrointestinal motility by GHWKCs corresponds to the TCM effect of “regulating stomach qi descending”; the anti-inflammatory and mucosal protective effects correspond to the TCM effect of “eliminating pathogenic factors and repairing damage”; the regulation of gut microbiota and bile acid metabolism corresponds to the TCM concept of “spleen governing transportation and transformation”. Notably, the identification of the Coriobacteriales-glycocholic acid axis as a putative core regulatory pathway provides a new mechanistic link between TCM theory and modern biology. Coriobacteriales is a beneficial bacterial order that plays a crucial role in bile acid metabolism, and its abundance has been reported to be reduced in patients with spleen deficiency syndrome. Our findings suggest that GHWKCs may exert its spleen-invigorating effect by restoring the abundance of Coriobacteriales and normalizing bile acid metabolism, which provides a scientific explanation for the TCM theory of “spleen governing transportation and transformation”.

This study provides preliminarily evidence for the therapeutic potential of GHWKCs through multidimensional analysis. However, several limitations should be acknowledged. First, orthogonal fingerprinting methods (e.g., HPLC-DAD, GC-MS) were not performed for GHWKCs chemical profiling. While the UPLC-HRMS method provides high-resolution mass spectral fingerprinting, future studies will incorporate multiple orthogonal techniques to strengthen quality control in accordance with ConPhyMP recommendations. Second, the high dose (1296 mg/kg/day) exceeded the recommended upper limit for rodent studies; however, the medium dose (648 mg/kg/day), representing the clinical equivalent, showed significant effects on all parameters and is considered the primary effective dose. Third, the correlation between *Coriobacteriales* and glycocholic acid does not establish causality; further studies using antibiotic depletion, fecal microbiota transplantation, or bile acid intervention are needed. Fourth, while we applied Benjamini–Hochberg FDR correction to all multi-omics datasets, we acknowledge that the FDR-adjusted threshold may be overly conservative for discovery-based untargeted metabolomics and microbiome studies. The use of FDR correction in such exploratory analyses remains a subject of ongoing debate, and we have interpreted our results with this consideration in mind. Finally, all findings are derived from a preclinical rat model, and well-designed clinical trials are required to confirm efficacy and safety in humans. Additionally, differences between animal models and human diseases restrict the direct application of these findings. Future research should focus on conducting rigorous clinical trials using refined evaluation methods to translate these fundamental discoveries into clinical applications.

In summary, this study systematically identified 63 chemical metabolites of GHWKCs using UPLC-LTQ-Orbitrap-MS analysis and established a rat model of FD that mimics the TCM pathogenesis of spleen deficiency and qi stagnation. Our results demonstrate that GHWKCs significantly improve the overall physiological status of FD rats through multiple mechanisms: enhancing gastrointestinal motility, alleviating mucosal inflammation, regulating gastrointestinal hormone levels, restoring bile acid metabolic homeostasis, and reshaping the gut microbiota structure. The integration of multi-omics analysis and network pharmacology revealed that the Coriobacteriales-glycocholic acid axis represents a core regulatory pathway of GHWKCs against FD, which bridges TCM theory of “spleen-stomach dysfunction” with modern molecular mechanisms. These findings provide comprehensive preclinical scientific evidence supporting the therapeutic potential of GHWKCs in FD treatment and offer new insights into the development of TCM-based therapies for functional gastrointestinal disorders. However, as all data were obtained from a rat model, further well-designed clinical trials are needed to confirm the efficacy and safety of GHWKCs in human FD patients.

## Data Availability

The datasets generated and analyzed during this study are available in the following public repositories: Metabolomics data: MetaboLights (accession number: MTBLS14314) (54); 16S rRNA sequencing data: NCBI Sequence Read Archive (accession number: PRJNA1454739); All network pharmacology raw data are provided in the Supplementary Materials of this article.

## Supplementary material

SUPPLEMENTARY TABLE S2 Detailed ADME parameters of the 31 active metabolites of GHWKCs.
